# Supplementary material for: Modeling glioblastoma heterogeneity as a dynamic network of cell states
Source: Mol Syst Biol. 2021 Sep 16;17(9):e10105. doi: 10.15252/msb.202010105 (PMC8444284; doi:10.15252/msb.202010105)
Supplement: Supplementary file 6 — Source Data for Figure 5 [file MSB-17-e10105-s004.zip › Figure5A_sourcedata/GSEA_3017/nosigdb_stateD.GseaPreranked.1622795010397/RICHARDS_NEFTEL_CELL_2019_G1_S.html]

Details for gene set RICHARDS\_NEFTEL\_CELL\_2019\_G1\_S[GSEA]

|  || Dataset | state23017 |
| Phenotype | NoPhenotypeAvailable |
| Upregulated in class | na\_neg |
| GeneSet | RICHARDS\_NEFTEL\_CELL\_2019\_G1\_S |
| Enrichment Score (ES) | -0.68435407 |
| Normalized Enrichment Score (NES) | -2.7645104 |
| Nominal p-value | 0.0 |
| FDR q-value | 0.0 |
| FWER p-Value | 0.0 |
Table: GSEA Results Summary

  

Fig 1: Enrichment plot: RICHARDS\_NEFTEL\_CELL\_2019\_G1\_S      
 Profile of the Running ES Score & Positions of GeneSet Members on the Rank Ordered List

  

| PROBE | GENE SYMBOL | GENE\_TITLE | RANK IN GENE LIST | RANK METRIC SCORE | RUNNING ES | CORE ENRICHMENT || 1 | MCM2 |  |  | 226 | -0.256 | -0.1573 | No |
| 2 | MCM3 |  |  | 625 | -0.305 | -0.4439 | No |
| 3 | MCM4 |  |  | 783 | -0.339 | -0.5425 | No |
| 4 | PKMYT1 |  |  | 795 | -0.342 | -0.5284 | No |
| 5 | CENPM |  |  | 818 | -0.349 | -0.5222 | No |
| 6 | TK1 |  |  | 902 | -0.375 | -0.5614 | No |
| 7 | CDC45 |  |  | 1000 | -0.413 | -0.6088 | No |
| 8 | ZWINT |  |  | 1099 | -0.467 | -0.6534 | Yes |
| 9 | ASF1B |  |  | 1133 | -0.498 | -0.6459 | Yes |
| 10 | PCNA |  |  | 1142 | -0.504 | -0.6187 | Yes |
| 11 | TMEM106C |  |  | 1148 | -0.509 | -0.5889 | Yes |
| 12 | GMNN |  |  | 1155 | -0.516 | -0.5593 | Yes |
| 13 | RRM1 |  |  | 1166 | -0.534 | -0.5317 | Yes |
| 14 | FEN1 |  |  | 1191 | -0.569 | -0.5126 | Yes |
| 15 | CKS1B |  |  | 1198 | -0.577 | -0.4790 | Yes |
| 16 | CENPK |  |  | 1220 | -0.619 | -0.4542 | Yes |
| 17 | DUT |  |  | 1223 | -0.633 | -0.4138 | Yes |
| 18 | CDCA5 |  |  | 1244 | -0.685 | -0.3839 | Yes |
| 19 | AURKB |  |  | 1250 | -0.700 | -0.3414 | Yes |
| 20 | MELK |  |  | 1251 | -0.701 | -0.2950 | Yes |
| 21 | RAD51AP1 |  |  | 1264 | -0.741 | -0.2553 | Yes |
| 22 | CCNE2 |  |  | 1273 | -0.771 | -0.2104 | Yes |
| 23 | RRM2 |  |  | 1295 | -0.933 | -0.1648 | Yes |
| 24 | HIST1H4C |  |  | 1320 | -2.769 | 0.0000 | Yes |
Table: GSEA details [plain text format]

  

Fig 2: RICHARDS\_NEFTEL\_CELL\_2019\_G1\_S: Random ES distribution      
 Gene set null distribution of ES for **RICHARDS\_NEFTEL\_CELL\_2019\_G1\_S**
